# Walking Faster and Farther With a Soft Robotic Exosuit: Implications for Post-Stroke Gait Assistance and Rehabilitation

**DOI:** 10.1109/OJEMB.2020.2984429

**Published:** 2020-04-02

**Authors:** Louis N. Awad, Pawel Kudzia, Dheepak Arumukhom Revi, Terry D. Ellis, Conor J. Walsh

**Affiliations:** ^1^ College of Health and Rehabilitation Sciences: Sargent CollegeBoston University 1846 Boston MA 02215 USA; ^2^ Harvard John A. Paulson School of Engineering and Applied SciencesHarvard University 1812 Cambridge MA 02138 USA; ^3^ Wyss Institute for Biologically Inspired EngineeringHarvard University 1812 Cambridge MA 02138 USA

**Keywords:** Exosuit, soft robotics, propulsion, walking speed, walking distance, stroke

## Abstract

*Objective:* Soft robotic exosuits can improve the mechanics and energetics of walking after stroke. Building on this prior work, we evaluated the effects of the first prototype of a portable soft robotic exosuit. *Methods:* Exosuit-induced changes in the overground walking speed, distance, and energy expenditure of individuals post-stroke were evaluated statistically and compared to minimal clinically important difference scores. *Results:* Compared to walking without the exosuit worn, the $< $5 kg exosuit did not substantially modify speed, distance, or energy expenditure when worn unpowered. In contrast, when powered on to provide an average 22.87 $\pm$ 0.58 %bodyweight of paretic plantarflexor force assistance during stance phase and assist the paretic dorsiflexors during swing phase to reduce drop-foot, study participants walked a median 0.14 $\pm$ 0.06 m/s faster during the 10-meter walk test and traveled 32 $\pm$ 8 m farther during the six minute walk test ($P < 0.05$). *Conclusions:* Individuals post-stroke can leverage the paretic plantarflexor and dorsiflexor assistance provided by soft robotic exosuits to achieve clinically-meaningful increases in speed and distance.

## I. Introduction

Portable robotic exoskeletons are the state of the art in wearable robotics [1]. These remarkable machines have enabled people who are unable to walk, to walk again. However, for people who retain the ability to walk after neurological injury, such as the majority of individuals post-stroke [2], rigid exoskeletons may not be necessary to restore more normal walking behavior [3]. Soft robotic exosuits made from garment-like functional textiles have emerged as a promising alternative [4]–[8].

Exosuits use a variety of approaches to deliver assistance during walking. Our prior work leveraged the interaction of cable-based transmissions and functional textile anchors to deliver mechanical power generated by actuators worn at the waist to a user's limbs during targeted phases of the gait cycle [9]. We have studied the effects of exosuits on the mechanics and energetics of post-stroke walking, demonstrating that exosuits can actively assist the paretic limb during treadmill walking to improve ground clearance, increase propulsion symmetry, reduce gait compensations, and reduce the metabolic burden of hemiparetic gait [4], [5], [10]. We have also shown that when used overground, exosuits can improve ground clearance and propulsion [5].

Although promising, the advance of soft robotic exosuits as either assistive or rehabilitation technology requires evaluating their effects on clinically-salient measures of walking ability. Clinical tests of short-distance walking speed (e.g., the 10-meter walk test) and long-distance walking ability (e.g., the 6-minute walk test) have strong prognostic value after stroke, accounting for a substantial amount of the variance in real world community walking behavior [11]–[13]. Moreover, improved performance during these timed walking tests would indicate more steps taken in the same amount of time, and thus a greater capacity to practice walking with higher repetition and intensity—treatment parameters that modulate the experience-dependent neuroplasticity that the effectiveness of rehabilitation is ultimately predicated on [14]–[17].

Building on our prior study of the effects of soft robotic exosuits on the mechanics and energetics of post-stroke walking [4], [5], [10], this exploratory study sought to evaluate the effects of a soft robotic exosuit on the short- and long-distance walking ability of individuals in the chronic phase of post-stroke recovery. To inform the translation of soft robotic exosuits to settings that may require walking with the exosuit powered off, a secondary objective was to evaluate the effects of an *unpowered* exosuit. We hypothesized that exosuit users with post-stroke hemiparesis would leverage the active assistance of paretic propulsion and ground clearance to walk faster and farther, whereas they would not change their walking behavior when walking with an unpowered exosuit.

## II. Results

Six individuals in the chronic phase after stroke participated in this study (Table 1). Study participants had a median (sIQR) age of 51.5 $\pm$ 9.9 years and were 4.2 $\pm$ 1.4 years post-stroke. Three of the six were female and four of the six were left hemiparetic. All study participants completed a screening visit and, to minimize the effects of fatigue within a session, two days of testing consisting of combined clinical and indirect calorimetry measurements (see *VI. Materials and Methods*). Clinical data were compared to minimal clinically important difference (MCID) scores (see *VI. D. Statistical and minimal important difference analyses)*. Day 1 included 10-meter walk test and 6-minute walk test assessments (i) without the exosuit worn and (ii) with an unpowered exosuit. Day 2 included the same assessments with the (i) unpowered exosuit and (ii) the exosuit powered and assisting paretic dorsiflexion during swing phase and plantarflexion during stance phase. Energy expenditure was measured during all 6-minute walk tests.

**TABLE 1.** Study Participant Characteristics.

Study Subject	Paretic Side	Sex	Age (y)	Chronicity (y)	Speed (m/s)
01	Right	F	30	7.08	0.86
02	Left	M	56	3.58	1.02
03	Left	F	52	0.75	0.67
04	Left	M	51	2.83	0.96
05	Right	M	29	6.08	0.87
06	Left	F	61	4.91	1.11

### A. Effects on Walking Distance

**Figure 1.**
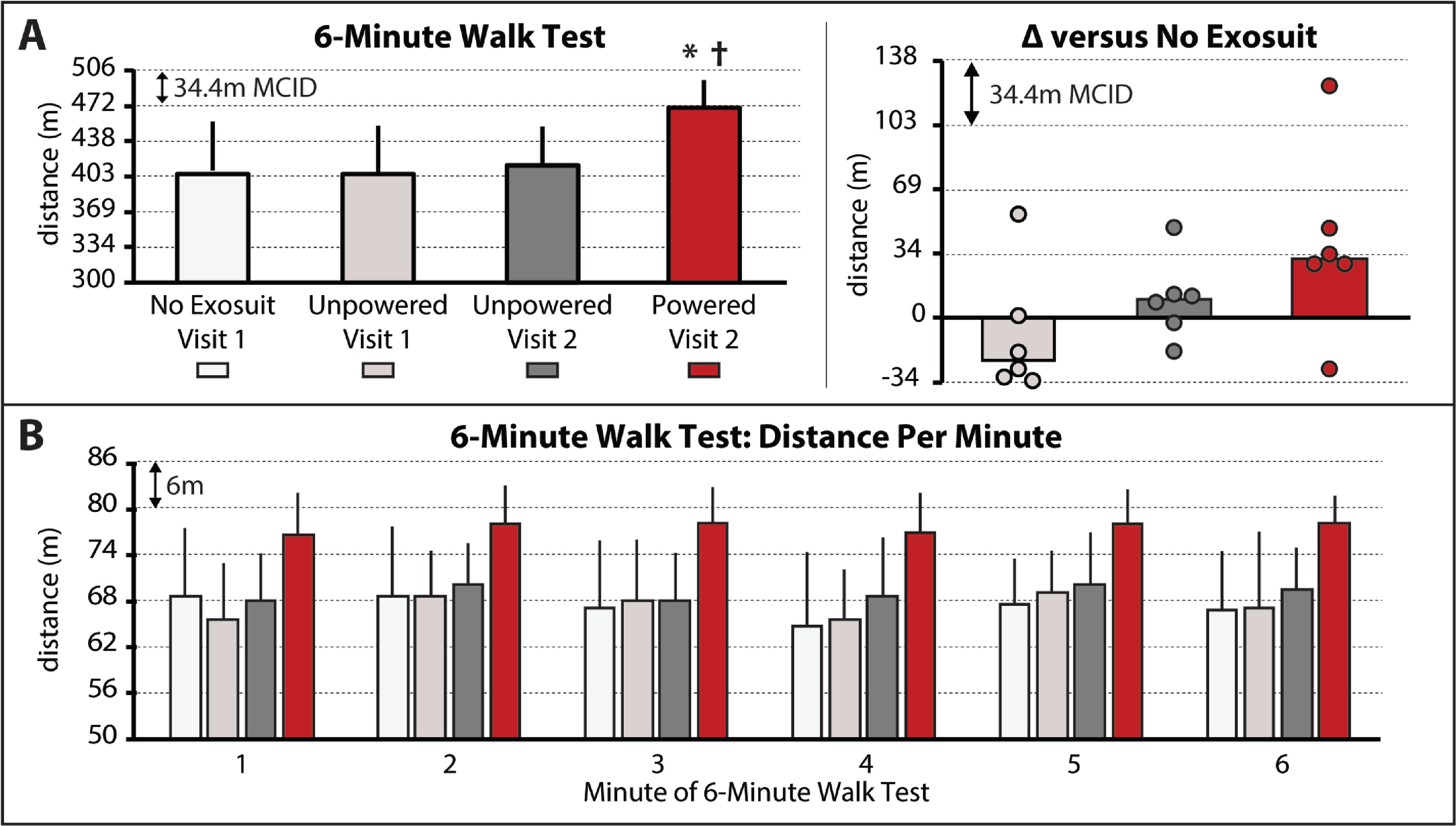
(A) Total 6-minute walk test (6MWT) distances for each condition. Right – Difference in 6MWT distance between the no exosuit condition and each of the two unpowered exosuit conditions and the powered exosuit condition. Circles represent individual study participants. (B) Distance per minute of the 6MWT for each condition. The minimal clinically important difference (MCID) is depicted in each plot with the dashed horizontal lines. Significance ($P < 0.05$) relative to $^\ast$no exosuit and $^\dagger$unpowered exosuit conditions. Medians and sIQR are reported.

The soft robotic exosuit influenced study participants’ walking distance during the 6-minute walk test ($\chi ^2(3) = 9.0, P = 0.029$) (see *Supplementary Material I. 6MWT Instructions*). Without the exosuit, study participants walked a median distance of 403 $\pm$ 49 m. With the unpowered exosuit, study participants did not significantly change their walking distance ($P > 0.05$), presenting with a 404 $\pm$ 45 m total distance during the unpowered exosuit test conducted on day 1 and a 412 $\pm$ 37 m total distance during the unpowered exosuit test conducted on day 2 (Fig. 1(A), Left). Interestingly, three of the six study participants approached a clinically meaningful reduction in walking distance during the first unpowered exosuit test conducted on day 1 (Fig. 1(A), Right); however, this penalty was not statistically significant nor was present on day 2. These findings suggest that there was some acclimation to walking with the exosuit unpowered across days and, together, indicate that an unpowered exosuit has a minimal effect on walking distance when compared to walking without an exosuit worn.

In contrast, compared to both the no exosuit and unpowered exosuit conditions, when the exosuit was powered on to provide targeted plantarflexor and dorsiflexor assistance, meaningful changes—both statistically ($P < 0.05$) and clinically (i.e., differences approximating the MCID)—in the distance walked were observed. More specifically, walking distance increased by a median 32 $\pm$ 8 m to 467 $\pm$ 26 m (Fig. 1(A), Left), with three of the six study participants surpassing the 34 m MCID and another two approaching the MCID by each increasing their walking distance by 29 m (Fig. 1(A), Right). Moreover, examination of the distance walked per minute revealed increases in distance during all minutes of the test (Fig. 1(B))—a finding of value in people post-stroke given the relationship between a distance-induced decline in walking speed and reduced real world walking activity [13].

### B. Effects on Walking Speed

**Figure 2.**
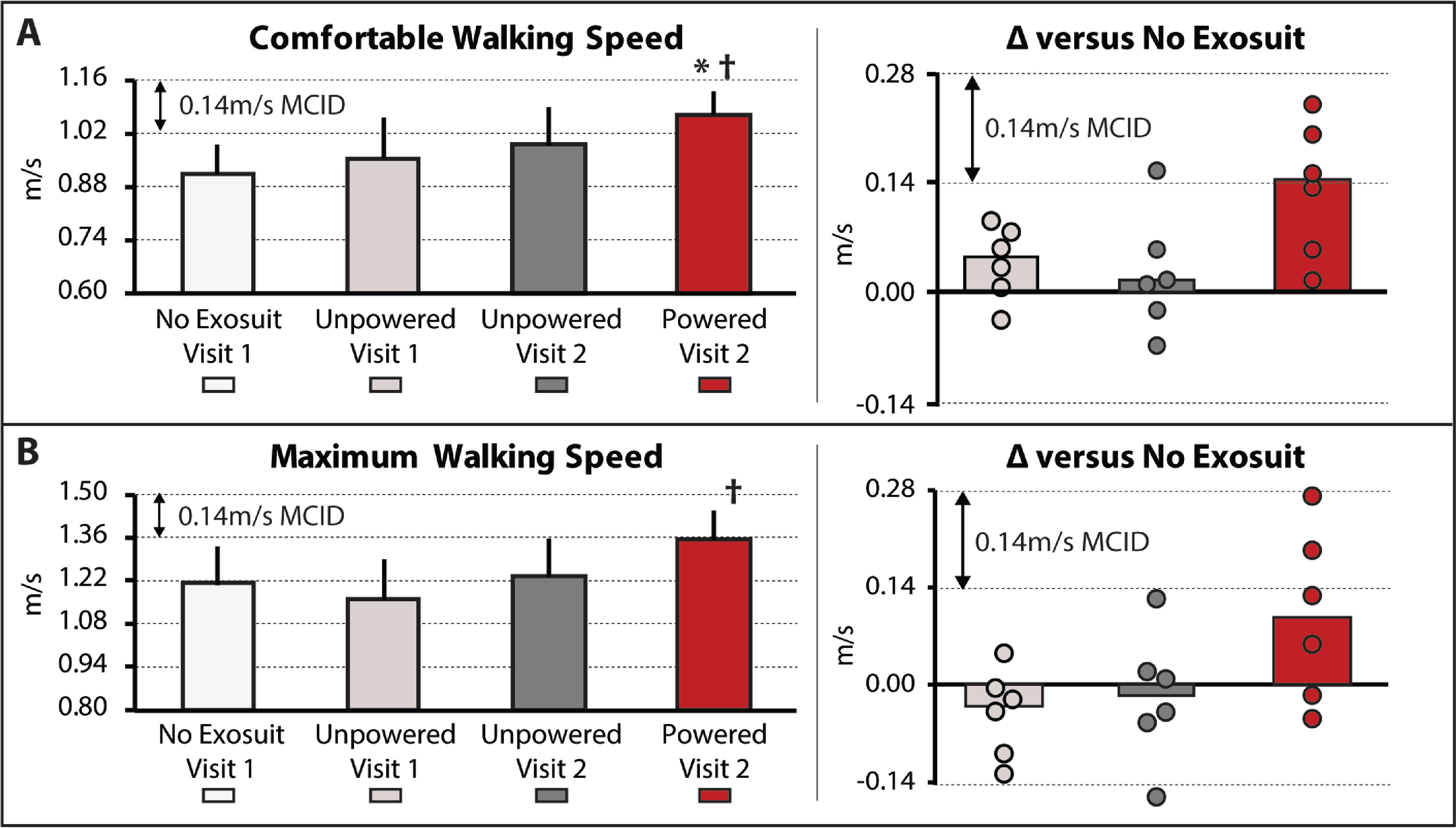
(A) Usual and (B) Maximum walking speeds, as measured using the 10-meter walk test, are shown for each condition tested. Right – The difference in each speed between the no exosuit condition and each of the two unpowered exosuit conditions and the powered exosuit condition. Circles represent individual study participants. The minimal clinically important difference (MCID) is depicted in each plot with the dashed horizontal lines. Significance ($P < 0.05$) relative to $^\ast$no exosuit and $^\dagger$unpowered exosuit conditions. Medians and sIQR are reported.

The soft robotic exosuit influenced study participants’ comfortable walking speed ($\chi ^2(3) = 8.4, P = 0.039$). Without the exosuit, study participants walked 0.91 $\pm$ 0.07 m/s. Consistent with the walking distance findings, when walking with the unpowered exosuit, significant changes in comfortable walking speed were not observed ($P > 0.05$), with study participants presenting with a 0.96 $\pm$ 0.10 m/s speed during the unpowered exosuit test conducted on day 1 and a 0.99 $\pm$ 0.09 m/s speed during the unpowered exosuit test conducted on day 2 (Fig. 2(A)). Moreover, as observed with the walking distance analyses, compared to both the no exosuit and unpowered exosuit conditions, when the exosuit was powered on, statistically ($P < 0.05$) and clinically (i.e., differences approximating the MCID) meaningful changes in comfortable walking speed were observed. More specifically, comfortable walking speed increased by a median 0.14 $\pm$ 0.06 m/s to 1.07 $\pm$ 0.06 m/s (Fig. 2(A), Left), with three of the six surpassing the 0.14 m/s MCID threshold and another approaching with a 0.13 m/s faster speed.

In contrast, maximum walking speed was not observed to be influenced by the soft robotic exosuit based on the omnibus test ($\chi ^2(3) = 6.2, P = 0.10$); however, given the exploratory nature of this proof-of-concept study, it is worth noting that the general response was consistent with the walking distance and comfortable walking speed findings. When compared to their 1.21 $\pm$ 0.12 m/s maximum walking speed while walking without an exosuit, study participants did not significantly change their maximum speed when walking with the exosuit unpowered ($P > 0.05$). Indeed, their day 1 unpowered exosuit speed was 1.16 $\pm$ 0.13 m/s and their day 2 unpowered exosuit speed was 1.24 $\pm$ 0.12 m/s. In contrast, when the exosuit was powered on, their maximum walking speed increased to 1.36 $\pm$ 0.09 m/s. The 0.09 $\pm$ 0.08 m/s median increase in maximum walking speed relative to walking without an exosuit was not statistically significant ($P = 0.12$); however, two of the six study participants surpassed the 0.14 m/s MCID and another two approached this threshold with increases of 0.13 m/s and 0.10 m/s. Moreover, when comparing the exosuit powered to unpowered conditions, the median increase of 0.13 $\pm$ 0.02 m/s was significant ($P = 0.028$). While promising, these exploratory findings for changes in maximum walking speed should be interpreted with caution given the non-significant omnibus test.

### C. Effects on Energy Expenditure During Walking

**Figure 3.**
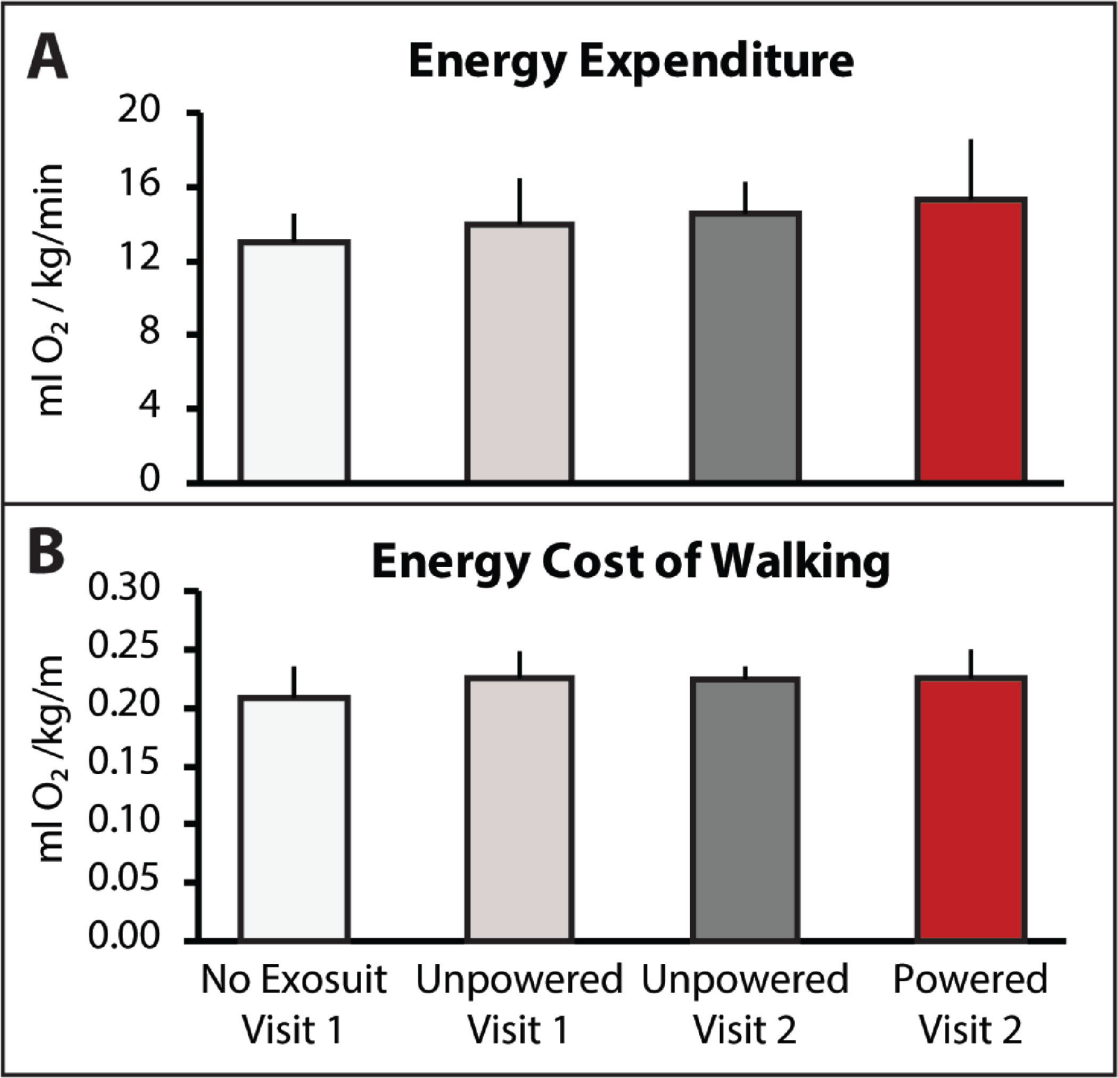
(A) Energy expenditure, measured with indirect calorimetry as oxygen utilization per kilogram of bodyweight and minute and (B) energy cost of walking, measured as oxygen utilization per kilogram of bodyweight and meter walked are presented for each condition. Medians and sIQR are reported.

The soft robotic exosuit was not observed to influence energy expenditure during walking ($\chi ^2(3) = 3.4, P > 0.05$). Without the exosuit, study participants walked with a median energy expenditure of 13.1 $\pm$ 1.5 ml O$_2$/kg/min. Consistent with the walking distance (Fig. 1) and speed (Fig. 2) findings, study participants did not significantly change their energy expenditure when walking with the unpowered exosuit ($P > 0.05$). Study participants' energy expenditure during day 1's test with the unpowered exosuit was 14.0 $\pm$ 2.4 ml O$_2$/kg/min and their day 2 energy expenditure with the unpowered exosuit was 14.6 $\pm$ 1.7 ml O$_2$/kg/min. Together, these findings suggest that an unpowered exosuit has a minimal effect on energy expenditure during walking (Fig. 3(A)).

Surprisingly, when the exosuit was powered on, despite study participants walking substantially farther (Fig. 1) and faster (Fig. 2), energy expenditure was not observed to significantly increase ($P > 0.05$) (Fig. 3(A)). That is, study participants appear to be able to use a powered exosuit to walk faster and farther without expending additional energy. However, an examination of differences in the energy cost of walking (i.e., speed-normalized energy expenditure) did not show differences across conditions ($\chi ^2(3) = 2.6, P > 0.05$) (Fig. 3(B))—perhaps due to a variable response across individuals, with one person reducing their energy cost of walking by 14%, another reducing by 5%, three having modest reductions $< $3%, and another increasing their energy cost by 18%.

## III. Discussion

In prior foundational biomechanical studies [4], [5], [10] we reported improvements in the mechanics and energetics of post-stroke hemiparetic walking with the targeted assistance of paretic plantarflexion and dorsiflexion by a soft robotic exosuit. In this exploratory clinical study, we report that people post-stroke can leverage soft robotic assistance to increase their overground walking speed and distance. These clinically-salient outcomes have strong associations to real world community walking [11], [12], patients’ perception of their ability to engage in home and community activities [18], and the amount of walking practice achieved during in-clinic rehabilitation [19]. By enabling people post-stroke to walk faster and farther, soft robotic exosuits may offer new opportunities for gait assistance and rehabilitation for those who do not benefit from existing devices [20], [21].

The immediate biomechanical benefits provided by soft robotic exosuits [4], [5], [10] and this study's demonstration of faster and farther walking with a soft robotic exosuit, when taken together, suggest that the technology may also have therapeutic value. Indeed, by helping patients walk faster and farther and with a better gait, exosuits have the potential to enable clinicians to provide task-specific walking practice of higher intensity and repetition—treatment parameters associated with better outcomes in those with neurological diagnoses [14], [22]. However, given the potential for neuromuscular slacking when walking with robotic assistance, and the association between neuromuscular slacking and reduced therapeutic outcomes [23]–[25], before the therapeutic value of soft robotic exosuits can be fully elucidated, the neuromuscular effects of walking with a soft robotic exosuit should be investigated.

A secondary objective of this study was to evaluate walking while wearing an unpowered robotic exosuit—i.e., the effects of the exosuit independent of the mechanical assistance it provides. Consistent with our prior work evaluating the effects of walking with just the textile portion of the soft robotic exosuit and no mechanical assistance [5], the changes in walking speed, distance, and energy expenditure induced by the $< $5 kg unpowered exosuit were neither clinically nor statistically meaningful. Although the small sample size of this exploratory study likely contributed to lower power for these analyses, our ability to detect significant and clinically meaningful changes in walking speed and distance when the exosuit was powered on in the same sample of study participants suggests that the effect of walking with an unpowered exosuit is smaller than the positive effects induced by the active assistance provided by the exosuit when powered on. A study with a larger sample size is necessary to confirm these exploratory findings.

People with post-stroke hemiparesis have been shown to complete timed long-distance walking tests with different strategies, with endurant individuals defined as those able to sustain their initial walking speed over the duration of the test and non-endurant individuals defined as those who reduce their walking speed by at least 0.10 m/s over the duration of the test [13]. Independent of the total distance that they are able to walk in six minutes, endurant individuals walk substantially more in the community than non-endurant individuals [13]. However, among endurant individuals, those who walk farther distances in six minutes present with more community walking activity than those who walk less total distances in six minutes [13]. All study participants in the present study would have been classified as endurant (i.e., any distance-induced decline in walking speed did not reach the 0.10 m/s threshold). Our finding that these higher functioning individuals were able to increase their long-distance walking speed during minute one of the 6-minute walk test by more than the 0.10 m/s threshold (i.e., increase the distance they walked during minute one by more than 6 m), and maintain this faster speed for the remaining five minutes, is thus noteworthy. In addition to supporting the translation of the exosuit technology as a community-based assistive device, this finding also supports the notion that soft robotic exosuits can increase the “dose” of walking practice achievable during a physical therapy session [26], and may thus have value as rehabilitation robots. Future study of the effects of a soft robotic exosuit on the long-distance walking performance of more impaired individuals post-stroke is warranted.

## IV. Limitations

A limitation of this study is that the no exosuit and powered exosuit conditions were tested on different days. This limitation is mitigated by our comparison of the differences between these conditions to established across-session MCID scores, as well as our assessment of walking with an unpowered exosuit on both days showing minimal differences. Relatedly, another potential limitation is that the testing order for each condition was fixed across study participants. This approach was chosen to streamline the experimental procedures, minimize fatigue, and avoid carryover of any neuromotor adaptations from the exosuit conditions to each day's baseline test; however, it is possible that an order effect may have been introduced. This limitation was, in part, mitigated by the forced rest breaks introduced between each of the two conditions tested on each day, wherein study participants were required to take a seated rest break until their vitals returned to their baseline levels. More importantly, examination of the first three testing conditions reported in Figs. 1 and 2 (i.e., no exosuit, exosuit unpowered 1, and exosuit unpowered 2) relative to the final testing condition (i.e., exosuit powered) demonstrates that any order effect introduced by this design was minimal relative to the marked effect of walking with the exosuit powered.

Other limitations of this study include the small sample size consisting of a high functioning cohort of individuals post-stroke and the inability to characterize the independent effects of the plantarflexion versus dorsiflexion assistance. Further study of these limitations, as well as the clinical potential of the exosuit technology, is warranted.

## V. Conclusions

Our prior studies reported that soft robotic exosuits can be used by individuals with post-stroke hemiparesis to improve paretic limb propulsion and ground clearance during treadmill and overground walking and reduce the energy cost of treadmill walking. In this study, we show that a portable robotic exosuit can be used by community-dwelling individuals with post-stroke hemiparesis to actively target plantarflexor and dorsiflexor deficits during overground walking in a manner that facilitates faster walking speeds and farther walking distances. Taken together, these exploratory findings suggest that exosuits may have substantial value as both community-based active assistive devices and in-clinic rehabilitation robots.

## VI. Materials and Methods

### A. Study Participants

A heterogeneous sample of six individuals post-stroke participated in this study (Table 1). The study inclusion criteria included the following: age between 25 and 75 years, at least 6 months after stroke, independent walking without an ankle foot orthosis, deficits in walking speed and distance, passive ankle range of motion with the knee extended to reach a neutral position, and passing the cognitive screening. Cognitive screening consisted of either a score $\geq$23 on the Mini Mental State Examination (MMSE) or, in persons with aphasia, a score $\geq$19 on the MMSE and a score $\geq$35 on the Auditory Verbal Comprehension section of the Western Aphasia Battery (WAB) and a score $\geq$10 on the Sequential Commands section of the WAB. Exclusion criteria included Botox treatments in the prior 6 months, substantial knee recurvatum during walking, comorbidities that prevented the safe completion of study procedures, an inability to communicate and/or be understood by investigators, a resting heart rate outside the range of 50 to 100 beats per minute or blood pressure outside the range of 90/60 to 200/110 mmHg, pain in the extremities or spine that limit walking, or a report of falling more than two times in the prior month. Medical clearance and signed informed consent forms approved by the Harvard University, Harvard Medical School, and Boston University institutional review boards were obtained for all study participants.

### B. Clinical and Metabolic Testing

After completing a screening visit confirming eligibility, study participants completed two days of overground walking evaluations consisting of both short and long-distance walking tests. The 10-meter walk test (10mWT) was used to evaluate short-distance walking speed and consisted of study participants completing three trials of walking 10 meters at a comfortable pace and three trials of walking 10 meters at a maximum pace. The middle six meters of the 10mWT was timed. Walking speed is reported in meters per second. The 6-minute walk test (6MWT) was used to measure long-distance walking function and consisted of study participants walking for six minutes (see *Supplementary Material I. 6MWT Instructions*). Walking distance was recorded with a digital measuring wheel by a research assistant closely following the study participant as they walked around a 30 m walkway. Walking distance is reported as the total distance walked during 6 minutes. The distance walked per minute of the 6MWT was also recorded. During each of the 6MWTs completed by study participants, a portable indirect calorimetry system (K4b2, Cosmed) was used to concurrently measure study participants’ energy consumption (ml O$_2$/kg/min). Energy consumption during the final three minutes of the 6MWT was normalized by the average walking speed during those minutes (m/min) to yield study participants’ energy cost of walking (ml O$_2$/kg/m).

On day one of testing, study participants completed the 10mWT and 6MWT evaluations first without the exosuit worn and then, after a required seated rest break, with the unpowered exosuit. On day two, study participants completed the same testing but first with the unpowered exosuit and then, after the rest break, with the powered exosuit. Although study participants could not use an ankle foot orthosis during testing, they could use their usual assistive device (i.e., cane) during testing if one was required for safety. If a cane was used during one test, for consistency, a cane was used during all tests.

### C. Exosuit Overview, Tuning, and Exposure

**Figure 4.**
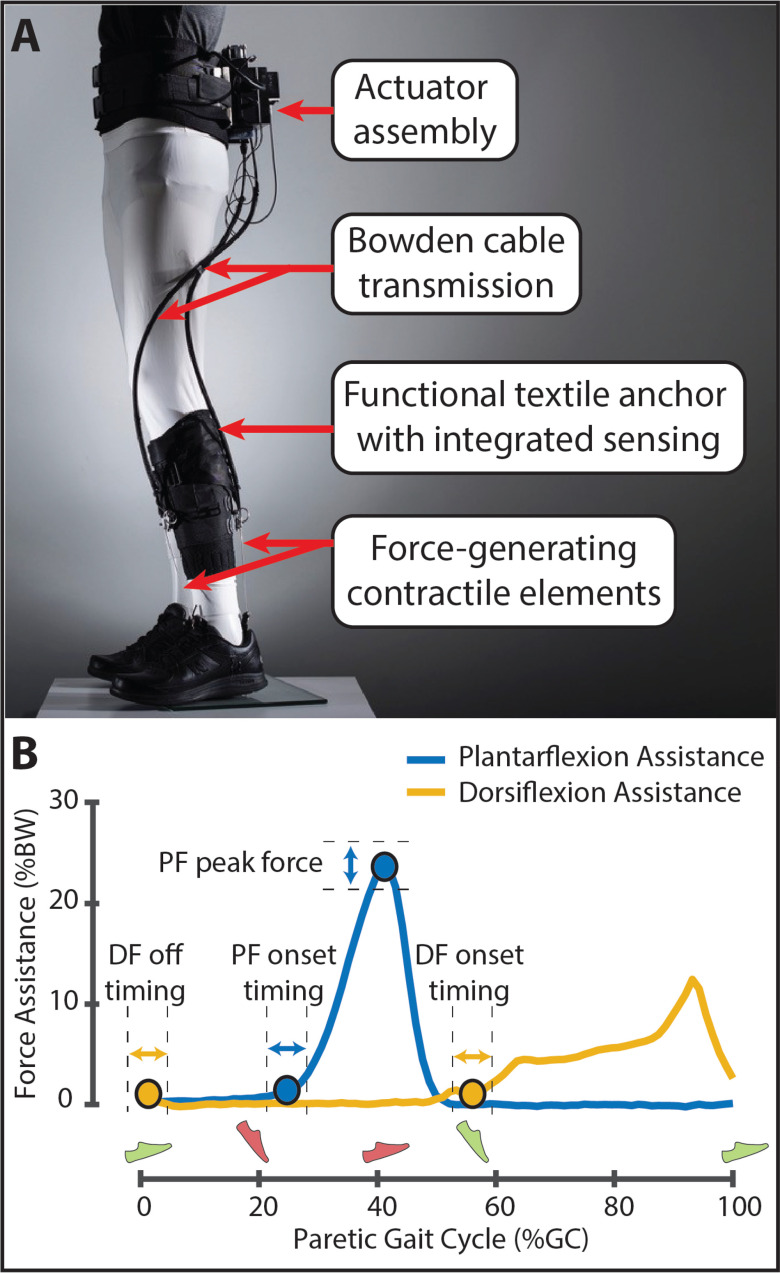
(A) Overview of the soft robotic exosuit used to augment paretic ankle plantarflexion (PF) and dorsiflexion (DF) function during post-stroke hemiparetic walking. (B) Exemplar force profiles for PF and DF assistance are shown. The onset timing and peak magnitude of PF assistance and the onset and off timing of DF assistance were commanded as described in previous work [5]. Assistance timing was delivered as a function of the paretic and nonparetic gait cycles defined by the detection of paretic (green) and nonparetic (red) foot strike and foot off. *Photo in panel A courtesy of Rolex Foundation.*

The soft robotic exosuit was designed to interface with the paretic limb of people post-stroke and has components worn proximally at the waist and distally on the paretic shank and shoe (Fig. 4(A)). The design is a portable adaptation of a previously studied tethered exosuit prototype [4], [5], [10], [27] and has been described previously [5]. In brief, the components worn at the waist weigh approximately 3.9 kg and consist of a mechanical actuator, battery, and the functional textile anchor used to securely attach the actuator and battery to the user. The components worn at the shank and shoe weigh approximately another 0.7 kg and consist of a sensor assembly containing load cell (Futek, Irvine, CA) and gyroscope (SparkFun Boulder, CO) sensors, a functional textile anchor worn around the shank to integrate the sensors and serve as the proximal attachment point for two Bowden cable assemblies, and a shoe insole that serves as the distal attachment point for each Bowden cable assembly.

Bowden cables transmit power generated by the mechanical actuator to the targeted ankle joint. Two Bowden cable assemblies were used, one located posterior to the ankle and designed to assist ankle plantarflexion during the paretic stance phase and one located anterior to the ankle and designed to assist ankle dorsiflexion during the paretic swing phase. As previously described [5], the retraction of the cables by the mechanical actuator generates the desired mechanical assistance during key periods of the gait cycle. More specifically, bilateral gait events are detected in real time using the gyroscopes worn on each shoe and used to control the initiation of plantarflexion assistance and the initiation and termination of dorsiflexion assistance. Moreover, the integrated load cells facilitate control over the magnitude of the peak plantarflexion assistance force and monitor the delivered dorsiflexion force (Fig. 4(B)). The magnitude of dorsiflexion force is not controlled, rather the magnitude of dorsiflexion cable retraction is set by a physical therapist based on visual observation of the amount of retraction needed to facilitate toe clearance by the paretic foot during its swing phase. As described in previous work, together, these sensors enable the exosuit to deliver appropriately timed and adaptive assistive forces [5], [27].

Summary statistics of the actual delivered plantarflexion and dorsiflexion forces are shown in Table 2. Across study participants, the median $\pm$ sIQR of the peak of the delivered ankle plantarflexion force was 23.19 $\pm$ 1.05 % bodyweight (%BW) and occurred at 43.44 $\pm$ 0.59 % of the gait cycle (%GC). Moreover, the median onset timing of plantarflexion assistance was 29.31 $\pm$ 1.58 %GC. The median onset timing of ankle dorsiflexion assistance was 55.16 $\pm$ 1.92 %GC and the median off timing was 4.29 $\pm$ 1.02 %GC.

Importantly, study participants were not instructed on how to walk with the exosuit; however, after day one's evaluations with the unpowered exosuit, study participants were provided with guided exposure to walking with the exosuit concurrent with the clinician-guided tuning of the exosuit's assistance parameters. More specifically, on day one of testing, after evaluating study participants’ 10mWT and 6MWT without the exosuit and with the unpowered exosuit, the device was then powered on and a physical therapist tuned the exosuit assistance to each user's walking pattern. Then, participants were instructed to complete 2-minute walking bouts at different walking speeds with the exosuit. These included bouts of (i) walking at their self-selected walking speed, (ii) maximum walking speed, and (iii) variable speeds ranging from slower than their usual speed to their maximum speed. A physical therapist provided guarding during this initial exposure to walking with an exosuit prior to the formal testing of the effects of walking with a powered exosuit on day two of the study.

**TABLE 2.** Exosuit Assistance Parameters.

Study Subject	Plantarflexion Assistance	Dorsiflexion Assistance			
	Peak Force (%BW)	*Peak Time (%GC)	Onset Time (%GC)	Onset Time (%GC)	Off Time (%GC)
01	-	-	-	-	-
02	23.2 $\pm$ 5.1	43.4 $\pm$ 5.4	30.1 $\pm$ 5.1	52.7 $\pm$ 6.3	7.3 $\pm$ 3.0
03	21.7 $\pm$ 5.3	42.4 $\pm$ 5.0	29.3 $\pm$ 4.1	55.2 $\pm$ 6.1	4.3 $\pm$ 1.7
04	23.8 $\pm$ 7.5	42.1 $\pm$ 6.6	27.0 $\pm$ 4.0	54.0 $\pm$ 5.7	0.7 $\pm$ 1.5
05	21.3 $\pm$ 4.4	44.5 $\pm$ 5.7	34.8 $\pm$ 5.0	59.6 $\pm$ 5.5	5.1 $\pm$ 2.8
06	24.2 $\pm$ 4.0	43.6 $\pm$ 3.0	26.8 $\pm$ 2.0	57.8 $\pm$ 4.5	3.0 $\pm$ 1.7

Participant 01's exosuit assistance data were not available due to technical issues. %BW – percent of bodyweight; %GC – percent of gait cycle. $^\ast$The peak timing of plantarflexion force was not a commanded exosuit parameter and was based on users’ kinematics. All other parameters were tuned to the individual.

### D. Statistical and Minimal Important Difference Analyses

Statistical analyses were conducted using the SPSS software package (IBM Inc, version 24). Alpha was set to 0.05. Nonparametric analyses were used given the small sample size. Walking distance and speed were the primary clinical outcomes of interest. Differences between the no exosuit, unpowered exosuit, and powered exosuit conditions were first evaluated using Friedman's omnibus tests. Post-hoc Wilcoxon signed rank tests were then conducted to test our primary hypothesis of differences between the powered exosuit vs. no exosuit conditions. Differences in walking distance were compared to a 34.4 m MCID for the 6-minute walk test based on the findings of [28] and similarly used by previous investigators to contextualize improvements in 6-minute walk test distance [29]–[32]. Differences in walking speed were compared to a 0.14 m/s MCID for the 10-meter walk test based on [33] and the midpoint of previously reported anchor-based MCIDs of 0.12 m/s [34] and 0.16 m/s [35]. Secondarily, to explore the effects of walking with an *unpowered* exosuit, pairwise comparisons of differences between the no exosuit vs. unpowered exosuit conditions were carried out for walking speed, distance, and energy expenditure. Finally, to inform future exosuit designs, differences between the powered exosuit vs. unpowered exosuit conditions were also tested and reported if a significant difference between the powered exosuit vs. no exosuit condition was not observed. We chose not to correct for multiple comparisons given the exploratory nature of this study, the non-independent nature of the comparisons, and the high degree of correlation across these variables (e.g., baseline performance across the timed walking tests was highly correlated, with Pearson r's $\geq$ 0.96) [36].

## Supplementary Materials

Supplementary material (Supplementary Material I. 6MWT Instructions) is included with this submission. A description of the methods used to evaluate the 6-minute walk test are included alongside a picture of a study participant completing the test while guarded by a research physical therapist.


