# Supplementary material for: Walking faster and farther with a soft robotic exosuit: Implications for post-stroke gait assistance and rehabilitation
Source: IEEE Open J Eng Med Biol. Author manuscript; Available in PMC 2021 Mar 18. (PMC7971412; doi:10.1109/OJEMB.2020.2984429)
Supplement: Supplementary file 1 [file NIHMS1592739-supplement-supp1-2984429.pdf]

Supplementary Materials

Walking faster and farther with a soft robotic exosuit: Implications for post-stroke gait assistance and rehabilitation

Louis N. Awad*, Pawel Kudzia, Dheepak Arumukham Revi, Terry D. Ellis, Conor J. Walsh

THE 6-minute walk test is a popular clinical outcome measure used across diagnostic groups to characterize functional walking capacity. The test was used in our study to evaluate the effects of the soft robotic exosuit on study participants' ability to sustain a fast walking speed for six minutes. The instructions provided to individuals who complete the test are known to influence their performance. For this study, we provided study participants with the following verbal instructions:

> "The object of this test is to walk as far as possible for 6 minutes. You will walk back and forth in this hallway. Six minutes is a long time to walk, so you will be exerting yourself. You will probably get out of breath or become exhausted. You are permitted to slow down, to stop, and to rest as necessary. You may lean against the wall while resting, but resume walking as soon as you are able. **Remember, the object of this test is to walk as far as possible for 6 minutes, and we will not stop the timer while you rest. You will be walking back and forth around the cones.** You should turn briskly around the cones and continue back the other way without hesitation. Now I am going to show you. Please watch the way I turn without hesitation."

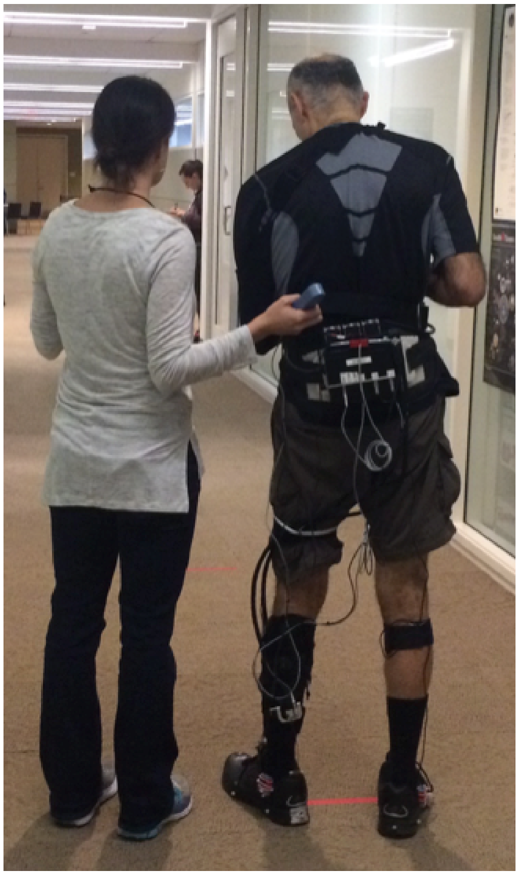

Fig. 1. (Supplementary) A study participant preparing to complete the 6-minute walk test.
